# Strain-Driven Dewetting and Interdiffusion in SiGe Thin Films on SOI for CMOS-Compatible Nanostructures

**DOI:** 10.3390/nano15130965

**Published:** 2025-06-21

**Authors:** Sonia Freddi, Michele Gherardi, Andrea Chiappini, Adam Arette-Hourquet, Isabelle Berbezier, Alexey Fedorov, Daniel Chrastina, Monica Bollani

**Affiliations:** 1 LNESS Laboratory, Institute of Photonic and Nanotechnology (IFN)-CNR, 22100 Como, Italy; 2 Department of Physics, Politecnico di Milano, 20133 Milano, Italydaniel.chrastina@polimi.it (D.C.); 3 Institute of Photonics and Nanotechnologies (IFN-CNR), CSMFO Laboratory and Fondazione Bruno Kessler (FBK) Photonics Unit, Via alla cascata 56/c, 38123 Trento, Italy; 4 IM2NP, Aix Marseille University, CNRS, F-13397 Marseille, CEDEX 20, France

**Keywords:** solid state dewetting, silicon germanium, nanoisland, nanostructures, strain relaxation, Mie resonators

## Abstract

This study provides new insight into the mechanisms governing solid state dewetting (SSD) in SiGe alloys and underscores the potential of this bottom-up technique for fabricating self-organized defect-free nanostructures for CMOS-compatible photonic and nanoimprint applications. In particular, we investigate the SSD of Si_1−x_Ge_x_ thin films grown by molecular beam epitaxy on silicon-on-insulator (SOI) substrates, focusing on and clarifying the interplay of dewetting dynamics, strain elastic relaxation, and SiGe/SOI interdiffusion. Samples were annealed at 820 °C, and their morphological and compositional evolution was tracked using atomic force microscopy (AFM), scanning electron microscopy (SEM), energy-dispersive X-ray spectroscopy (EDX), X-ray diffraction (XRD), and Raman spectroscopy, considering different annealing time steps. A sequential process typical of the SiGe alloy has been identified, involving void nucleation, short finger formation, and ruptures of the fingers to form nanoislands. XRD and Raman data reveal strain relaxation and significant Si-Ge interdiffusion over time, with the Ge content decreasing from 29% to 20% due to mixing with the underlying SOI layer. EDX mapping confirms a Ge concentration gradient within the islands, with higher Ge content near the top.

## 1. Introduction

Solid-state dewetting (SSD) is a phenomenon in which a thin film undergoes morphological changes, breaking up into isolated nanostructures. This process occurs when the film becomes unstable, due to for instance a thermal treatment in ultra-high vacuum (UHV). SSD is driven by energy minimization (resulting from the balance between surface and interfacial energies); it can occur below a film melting temperature [1,2], and it is mainly driven by mass transport mediated by surface diffusion: atoms on the film surface migrate away from the edges, causing retraction, bulging, the formation of fingers, and ultimately the fragmentation of the film into isolated nano-islands [3,4,5]. Film thickness, as well as annealing time and temperature, are the main parameters that influence the island shape and size [6,7,8,9,10,11].

Dewetting has become more controllable in recent years, and several applications of dewetted materials have been developed, including photodetection [12,13,14,15], photocatalysis [15,16], Mie resonators [17,18], microfluidic channels [19,20], bacteria detection [21,22], and localized surface plasmon resonator sensors [23].

Additionally, nanostructures obtained by SSD can be exploited as hard masters for nanoimprint lithography (NIL) processes [24,25], offering exceptional pattern fidelity and the possibility of producing complex nanoscale patterns with low cost and high throughput.

Crystalline silicon-on-insulator (SOI) thin film represents the class of material most exploited to study dewetting dynamics, and it is considered to be a model for SSD investigations [26,27,28,29]. More recently, the SSD of silicon-germanium alloys (SiGe) has raised the interest of researchers, mainly due to the possibility of exploiting them in a wider range of applications. Indeed, SiGe is more responsive in the infrared spectrum compared to pure Si, making it useful for photonic applications such as photodetectors, modulators, sensors and integrated optical circuits [30,31,32,33,34]. SiGe nanostructures, such as quantum dots or wells, can be engineered to achieve enhanced light emission compared to pure Si, addressing a key limitation in silicon photonics [35]. Additionally, SiGe is compatible with existing silicon manufacturing processes, enabling easy integration into standard CMOS technologies [36,37].

Several top-down fabrication techniques are commonly employed to create Si and Ge nanocrystals from SOI and GeOI substrates. Electron beam lithography (EBL) and NIL enable high-resolution patterning followed by anisotropic etching to define nanoscale features with precise control over size and shape [38,39,40]. Focused ion beam (FIB) milling allows direct material removal for the rapid prototyping of nanostructures, but may induce surface damage that requires post-annealing [41]. Block copolymer lithography offers a cost-effective route to densely packed nanoscale patterns through self-assembled polymer templates [42,43]. Finally, nanocrystals of SOI and GeOI can be grown using epitaxial methods, such as Molecular Beam Epitaxy (MBE). This approach allows for precise control over crystal size, composition, and spatial distribution. In particular, MBE offers atomic-scale accuracy in layer-by-layer deposition, which is advantageous for forming defect-free nanocrystals on insulating substrates [44,45,46,47]. However, the lattice mismatch between nanocrystals and the underlying oxide layer, along with the low diffusion rates on insulating surfaces, present significant challenges that require the careful optimization of growth conditions, including substrate temperature, flux rates, and surface preparation. While these methods provide excellent pattern fidelity and scalability, they often involve complex processing steps and risk introducing defects during etching or ion exposure, which can affect the crystalline quality of the resulting nanostructures.

In contrast, as an alternative to the aforementioned top-down fabrication techniques and epitaxial growth methods, there are many advantages of nanostructure fabrication exploiting dewetting. These include the capability to produce atomically flat nanostructures, and the better scalability of the process compared to other approaches [6].

The present work focuses on the intrinsic evolution of strain relaxation and interdiffusion during spontaneous SSD, providing a foundation for future investigations into guided or templated dewetting strategies. As mentioned, several studies have demonstrated that parameters such as film thickness, annealing temperature, and duration significantly influence the size and density of the resulting islands [6,11,29,48], while pre-patterning the substrate or film surface using top-down approaches, such as EBL, NIL, or templated etching, can guide the dewetting process and enable ordered nanostructure arrays [17,18,49]. In detail, we investigate the SSD of a silicon–germanium (Si_1−x_Ge_x_) thin film deposited on a SOI layer, with the aim of clarifying the sequence and interplay of key phenomena occurring during annealing: SSD, morphological relaxation, and Si diffusion into the dewetted nanostructures. By carefully tuning annealing conditions and monitoring the structural evolution of the films, we aim to identify which of these processes initiates first, and how they influence each other, exploiting several characterization techniques.

The composition x and in-plane strain ε_‖_ of the Si_1−x_Ge_x_ layers were measured using high-resolution X-ray diffraction (XRD), employing the (004) and grazing incidence (224) Bragg reflections. The morphology is characterized in detail using scanning electron microscopy (SEM) and atomic force microscopy (AFM). A correlation between the XRD analysis and Raman spectroscopy data demonstrates the composition evolution in Si_1−x_Ge_x_ alloys during the annealing process, due to intermixing between the SiGe and SOI layer as well as the relaxation of strain.

Our study sheds light on the fundamental dynamics governing SiGe thin film stability and provides valuable insights for optimizing the dewetting-based fabrication of nanostructures for photonic and nanoimprint applications.

## 2. Materials and Methods

First, 300mm commercial SOI wafers (12 nm Si “device” layer on 25 nm SiO_2_ BOX on Si(001) “handle” substrate) were cut into square 2 cm × 2 cm samples and cleaned with a chemical treatment before introduction into the molecular beam epitaxy (MBE) chamber (Marseille, France). Then, 15 nm of Si_0.71_Ge_0.29_ was grown using solid sources (electron beam evaporator for silicon and effusion source for germanium).

To avoid surface contamination, samples were cleaned before the annealing. In detail, samples underwent sonication in acetone for 10 min, ringing in isopropanol and drying with N_2_ flux, and then, to eliminate potential organic contamination, a plasma Asher was performed (500 W, 500 L/min, 10 min). No sign of surface contamination was detected in the morphological or compositional characterizations.

The dewetting of the SiGe layers is performed by heating the sample in UHV conditions in a dedicated custom-made annealing machine, which can reach a pressure down to 10^−10^ mbar and a temperature increment rate of 40 °C/min. Temperatures up to 900 °C can be reached with a halogen lamp (maximum power: 400 W), and an automatic controller managed the ramp used to achieve the desired temperature [6].

The first step of the annealing process is represented by outgassing, performed at 500 °C for 30 min at 10^−9^ mbar, in order to evaporate impurities and water from the sample surface. After outgassing, to study the evolution of the dewetting process, annealing treatments were carried out at 820 °C, varying the time of annealing: 10 min (sample A), 15 min (sample B), 20 min (sample C), and 30 min (sample D).

The AFM images were acquired in tapping mode, using a super-sharp silicon probe (typical radius of curvature 2 nm), and processed with align rows-median to remove skipping lines using the Gwyddion software (version 2.54).

SEM imaging was performed using a Jeol system (JSM IT800(IS), Milan, Italy), operating at 5 kV, while the EDX analysis was performed at 20 kV.

X-Ray diffraction (XRD) was performed with a PANalytical (Almelo, The Netherlands) X-Pert ProMRD high-resolution diffractometer equipped with a Cu Kα source and hybrid monochromator, to investigate the SiGe alloy composition of the samples as well as to probe their strained/relaxed nature.

To further probe the strained/relaxed nature of the samples, Raman spectroscopy was performed using a LabRAM Aramis (Kyoto, Japan), equipped with a 633 nm laser source. The laser light was focused on the samplewith a 100× objective, and a 1800 l/mm grating and a 60 μW laser power used to avoid sample heating.

## 3. Results

**Figure 1:**
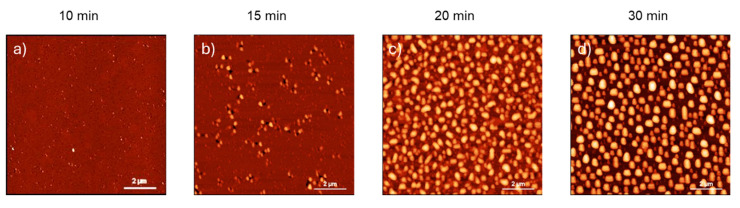
AFM topography images acquired on the samples annealed at 820 °C for (**a**) 10 min, (**b**) 15 min, (**c**) 20 min, (**d**) 30 min. The scale bar is the same for all images (2 μm).

To track the changes in film roughness during various annealing stages, AFM measurements were carried out (Figure 1). In the first 10 min of annealing, the layer showed an increase in the root mean square roughness (RMS) from about 0.10 nm (as grown sample) to 0.35 nm (Figure 1a). The RMS further increased up to 5.2 nm (Figure 1b), as the annealing time continued, as can be expected. After 15 min of annealing, a substantial change in the morphological features of the surface was observed: while voids appeared (dark spots), features with a greater height also started to form (bright spots) around voids. After 20 min of annealing, the surface presented bright structures, which were mainly connected (Figure 1c), whereas after 30 min of annealing, the surface was covered with isolated structures (Figure 1d). Regardless of the specific temperature at which the dewetting instability occurs, the variation in roughness and the subsequent rupture of the film at the interface are characteristic of a typical dewetting process in silicon-based alloys [8,17,18].

**Figure 2:**
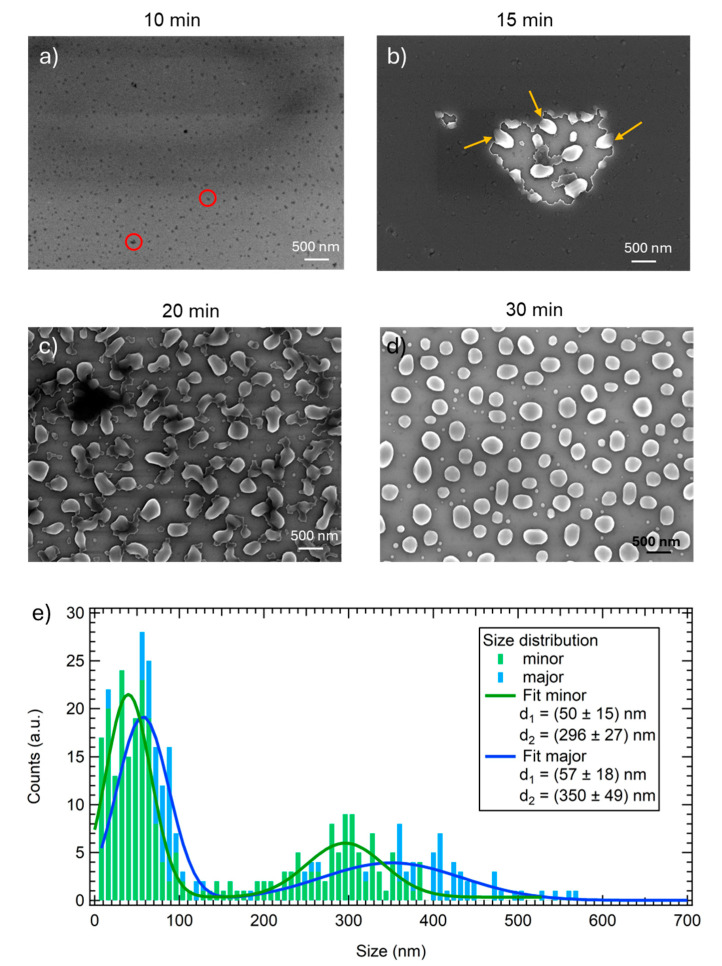
SEM images acquired in planar view after annealing for (**a**) 10 min, (**b**) 15 min, (**c**) 20 min, (**d**) 30 min. (**e**) Size distributions of the SiGe islands for the minor and major axes, evaluated considering several SEM images acquired on the 30 min annealed sample. The key reports the mean values of the bimodal distributions. Red circles in panel (**a**) indicate voids, while yellow arrows in panel (**b**) indicate the short fingers formed during the SSD process.

SEM investigation offers more insight into the morphology evolution during annealing. The as-grown surface is completely flat and does not present defects (not shown here). After 10 min of annealing at 820 °C, the surface appears rougher, in agreement with the increase in the RMS value observed by AFM measurements, and it is possible to observe the appearance of random voids (dark spots in Figure 2a; for clarity, a couple of them are highlighted with red circles), which were not clearly visible with AFM (Figure 1a). Therefore, spontaneous dewetting starts with the heterogeneous nucleation of voids forming randomly, followed by the opening of the voids and the formation of elongated structures, called fingers, as can be seen in Figure 2b (yellow arrows). The fingers then break up, creating isolated structures inside the voids.

Upon a further increase in the annealing time, the voids expand across the whole sample area and the dewetting proceeds, creating connected structures (Figure 2c) which gradually become more separated and circular, until reaching the formation of the complete dewetted nano-islands shown in Figure 2d. As can be observed by the SEM image, the dewetted nanoislands are not perfectly spherical; indeed, the mean value for the circularity of the structures was found to be 0.76 ± 0.02. The size distribution of the dewetted nano-islands in the 30 min sample is reported in Figure 2e.

A bimodal distribution is observed, in agreement with what has been already reported in the literature for dewetted systems of a-Ge [6,7], a-Si [37], and also for SiGe alloy [50] films; therefore, the data have been fitted with two Gaussian functions. Considering the 0.76 circularity value, two distributions are reported: one for the minor axis (green curves) and one for the major axis (blue curves). The smallest nano-islands are more circular, since the minor and major axes’ mean values are quite close: (50 ± 15) nm and (57 ± 18) nm, respectively.

**Figure 3:**
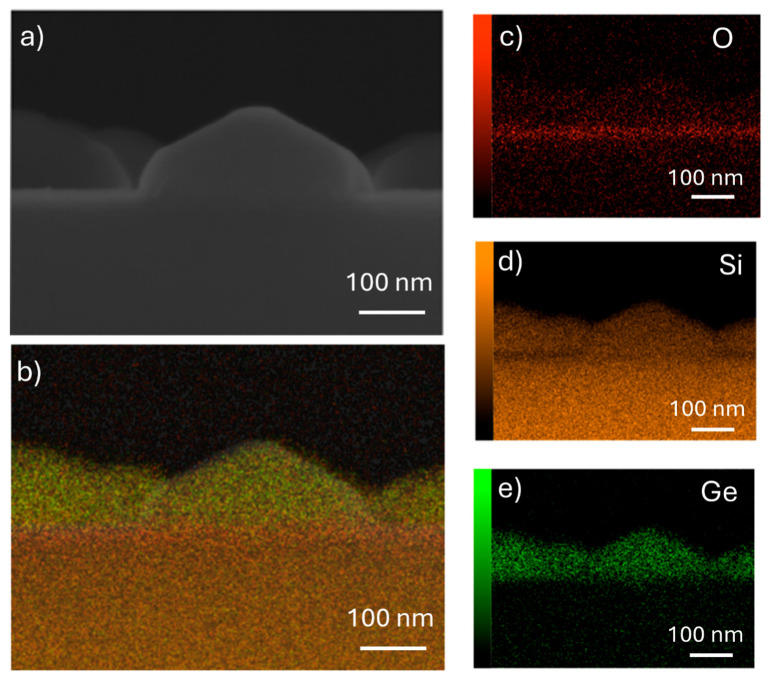
SEM image acquired in cross view (90°) on the 30 min annealed sample with corresponding EDX mapping. (**a**) SEM cross section image; (**b**) sum of chemical elements present in the sample: red is oxygen, orange is silicon, and green is germanium. Single element maps: (**c**) oxygen; (**d**) silicon; (**e**) germanium.

To investigate the chemical composition of the islands, EDX mapping and spectra are acquired in cross view on the 30 min annealed sample (D sample). In Figure 3a, a side-on image of an island is reported: facets confirming the crystalline nature of the nanostructures formed by SSD are clearly visible. Regarding the chemical composition, firstly, the maps reported in Figure 3 confirm the presence of germanium, oxygen, and silicon; in particular, oxygen is mainly present in the silicon buried oxide (BOX) layer underneath the islands, germanium is found only in the islands, and silicon can be found on the whole map. Nevertheless, the intensity of the silicon contribution is different: it is more intense in the BOX and in the substrate, while a lower intensity is detected in the islands, indicating a lower concentration, and the SOI layer cannot be distinguished.

**Figure 4:**
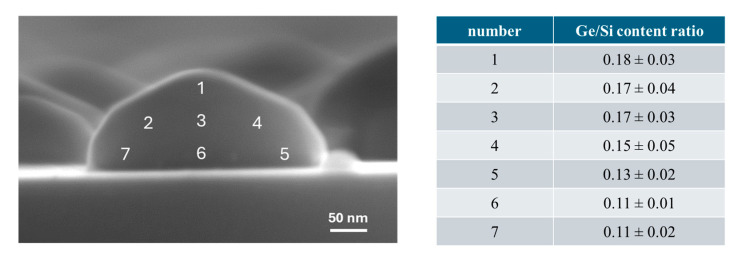
Representative SEM image acquired in cross view (90°) on a nanoisland of the 30 min annealed sample. In the SEM images, the numbers indicate the area where single spectra have been acquired, and the quantification analysis for this is reported in the table on the right side. It can be seen that more Ge is generally present towards the top of the nanoisland.

To better understand whether different Ge content is present inside the islands, single spectra have been acquired in different regions of several islands, and the relative Ge/Si composition results obtained by a statistical analysis are reported in Figure 4. From this analysis, it is clear that a gradient is present. The islands are richer in Ge at the top, while at the bottom the Ge content is lower. This is in agreement with an interdiffusion process of silicon from the SOI layer, which has been already observed in the literature [51].

This gradient in Ge content is observed in all the dewetted islands, independent of the size. Nevertheless, it is fair to point out that in the case of smaller islands (diameters up to 80 nm), the spatial resolution of SEM/EDX may introduce a not-negligible error in the spectra acquisition, and the Si substrate and the surroundings may alter the qualitative evaluation of the Ge/Si ratio.

The average Ge composition *x* of the Si_1−x_Ge_x_layer is also obtained from the XRD acquisition of symmetric (004) and asymmetric (224) reciprocal space maps. Starting from the as-grown layer, where the Ge content *x* = 0.29, at 10 and at 15 min of annealing, the composition remains similar at *x* = 0.29. For sample C, after 20 min of annealing, the composition drops to 0.22 and then to 0.20 after 30 min, as the SiGe layer intermixes with the Si device layer of the as-grown sample. In sample C we note that the X-ray beam illuminates regions of the sample at different stages of dewetting.

**Figure 5:**
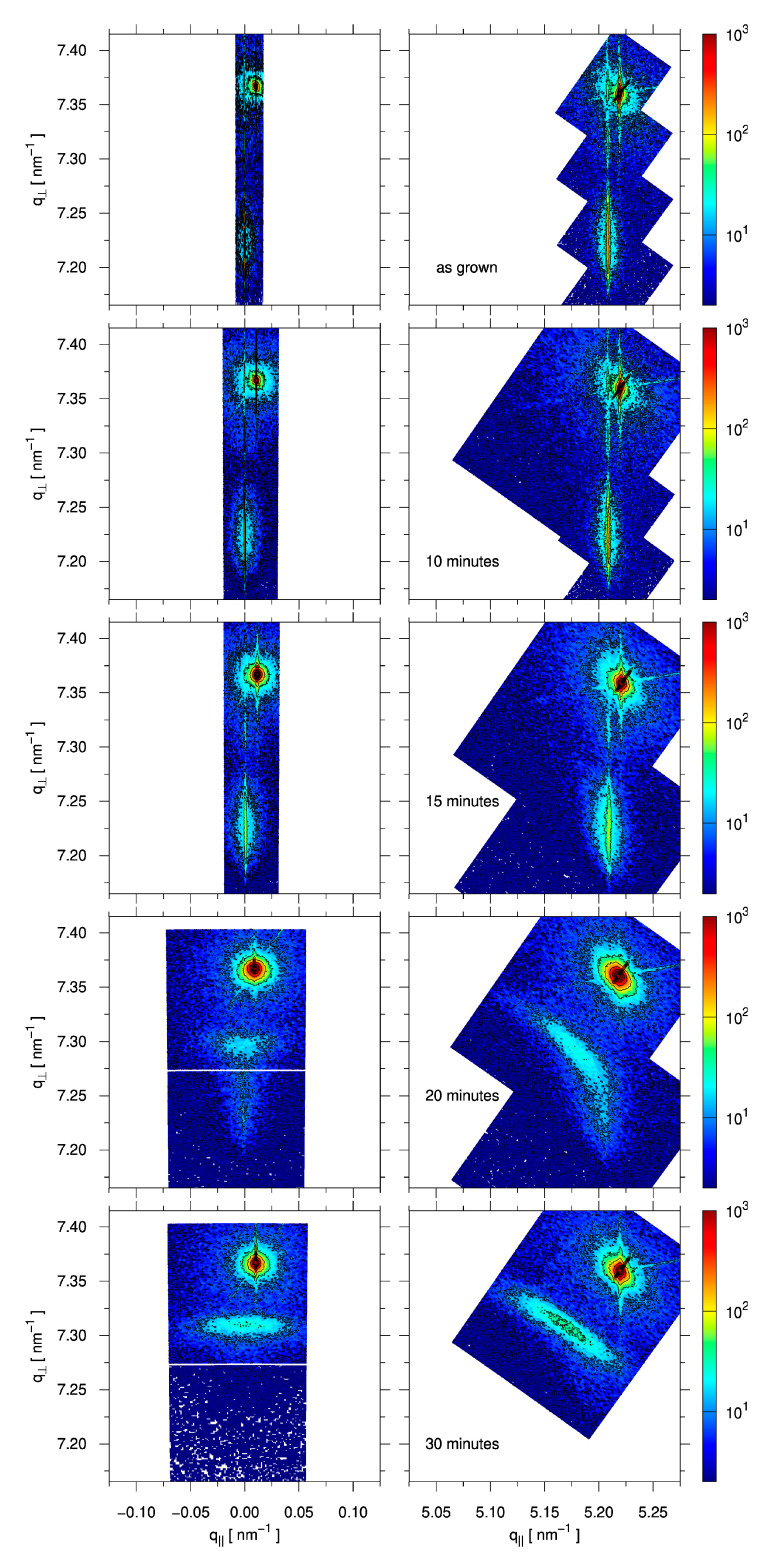
Reciprocal space maps (RSMs) around the (004) (**left**) and (224) Bragg peaks. The sharp Si bulk peak can be seen in the top right. The thin SOI “device” layer is slightly misaligned with respect to the “handle” substrate. The epitaxial SiGe layer is well-aligned to the device layer, as is especially clear in the first three samples (as-grown, 10 min, 15 min). No appreciable strain relaxation occurs up to 15 min, but some broadening of the SiGe peak occurs after 15 min due to the introduction of the first defects. At 20 min, the formation of a relaxed SiGe layer containing less Ge can be seen, and at 30 min the whole film is relaxed.

XRD also provides valuable information on the strained or relaxed nature of the samples (Figure 5).

In the reciprocal space maps (RSMs), the sharp peak at *q*_⊥_ = 4/*a*_Si_ = 7.37 nm^−1^ corresponds to the Si “handle” substrate [52]. The alignment of the RSMs has been corrected such that the thin SOI “device” layer is at *q*_‖_ = 0.00 nm^−1^ in the (004) RSMs. The Si_1−x_Ge_x_layer is then seen at lower *q*_⊥_ values, since Si_1−x_Ge_x_generally has a larger lattice parameter than Si. In the case that the Si_1−x_Ge_x_is fully strained, its in-plane lattice parameter *a*_‖_ will match that of Si. In the (224) RSM, this means *q*_‖_ = √(2^2^ + 2^2^)/*a*_Si_ = 5.21 nm^−1^. Due to a Poisson ratio of about 0.277, this in-plane compression is associated with expansion in the growth direction, moving the Si_1−x_Ge_x_peak to an even lower *q*_⊥_ value. A fully relaxed Si_1−x_Ge_x_layer would instead be found in the (224) RSM at *q*_‖_ = √(2^2^ + 2^2^)/*a*_0_(*x*) and *q*_⊥_ = 4/*a*_0_(*x*), in which *a*_0_(*x*) is the natural lattice parameter of the cubic Si_1−x_Ge_x_alloy. Relaxation is also associated with the introduction of defects between the Si_1−x_Ge_x_and Si layers, which causes a broadening of the peaks [53].

**Figure 6:**
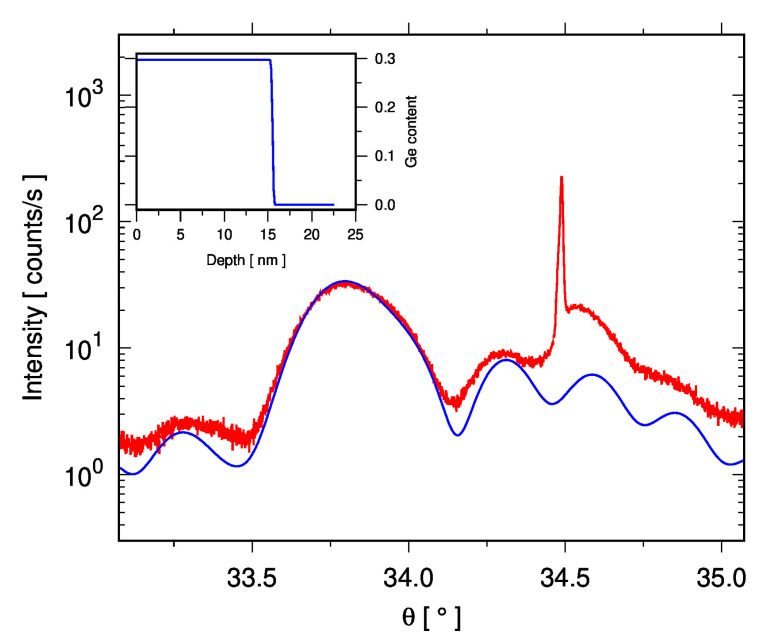
Double-crystal (004) ω-2θ scan (red curve) of the “Flat” as-grown sample, showing the broad SiGe peak at θ = 33.6° with intensity from the Si “device” and “handle” substrate around θ = 34.5°. Note that the peak from the handle is weak and shifted in position since it is not exactly in the Bragg condition (there is about 0.1° tilt between the (001) planes of the device and handle). The inset shows the Ge profile used to calculate the dynamical Darwin model simulation (blue curve).

The as-grown sample is fully strained, as can be expected, with *ε*_‖_ = −1.10. An ω-2θ double-crystal scan through the (004) peaks of the Si_1−x_Ge_x_layer and Si “device” layer of the as-grown sample is shown in Figure 6, along with a dynamical simulation calculated using the Darwin model from the xrayutilities package [54], which confirms the thickness of the SiGe layer.

In Figure 5, it can be seen that full strain remains after 10 min of annealing (sample A). For sample B, i.e., after 15 min of annealing, the SiGe peak starts to become wider, as can be seen in Figure 5, without significant relaxation. It is only after 20 min of annealing that relaxation starts (*ε*_‖_ = −0.90 and *ε*_‖_ = −0.22, in the two different areas considered), leading to sample D being close to full relaxation after annealing for 30 min (*ε*_‖_ = −0.05).

**Figure 7:**
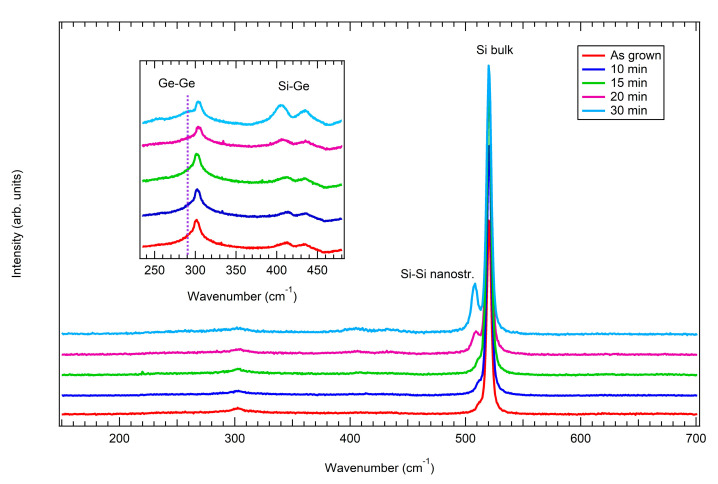
Raman spectra collected on the flat sample (red curve) and on the annealed samples: 10 min (blue curve), 15 min (green curve), 20 min (purple curve), and 30 min (light blue curve). The insert presents a zoomed-in view of the wavenumber region between 240 and 465 cm^−1^.

Raman spectra collected on the samples are reported in Figure 7. The insert presents a zoomed-in view of the region between 240 and 465 cm^−1^, in which the intensity is much lower compared to the main Si-Si peak of the substrate at around 520.5 cm^−1^.

Let us start by considering the flat sample (red curve). The presence of the main Si-Si peak related to the substrate is detected at around 520.5 cm^−1^, while a shoulder at 510.8 cm^−1^ is ascribed to the Si-Si peak in the presence of nanostructures. Furthermore, the second order of the Si-Si mode (2TA) is seen at around 300 cm^−1^. The SiGe contribution is in the region between 400 cm^−1^ and 450 cm^−1^, with the presence of two peaks: the Si-Ge vibrational modes at about 412 cm^−1^ and the localized Si–Si modes at about 437 cm^−1^. The localized Si–Si mode is related to a change in the local symmetry of the SiGe alloy, which results from the random introduction of Ge atoms into the Si lattice [55]. Finally, a shoulder of the 2TA silicon mode, at 290 cm^−1^, indicates the presence of the Ge-Ge vibrational mode. Its intensity is particularly low due to the low Ge concentration in the SiGe layer.

Starting with annealing and increasing the annealing time, all the peaks remain present in the samples, even if there are some differences: the peak around 510.8 cm^−1^ appears downshifted and more intense on increasing the annealing time, as do the Si-Ge and Ge-Ge vibrational modes. This is related to the relaxation of the strain of the samples as well as of the reducing Ge content, as seen by XRD, and that will be further analyzed in the Section 4 of the manuscript.

## 4. Discussion and Conclusions

As mentioned, the dewetting features of SiGe alloys have been studied less compared to their silicon counterparts. Comparing the two systems, it is clear that SiGe dewetted areas do not present the typical regular square shape of silicon (formed in the [110] and [111] directions) and that voids tend to assume a more rounded shape with an irregular perimeter (Figure 2b), lacking a visible long-range order along the [130] direction which can be easily found in Si samples. The presence of fingers is still noticed (Figure 2b), although they are shorter compared to the ones formed on silicon.

These results are in agreement with what has been reported in the literature [50,56,57]. In particular, Zhang et al. [57] evidenced how the presence of Ge destroys the long-range ordering of the Si islands formed during the dewetting process.

In detail, to summarize the experimental evidence reported in the Section 3, first the appearance of voids is observed, followed by rim retraction and finger formation. Proceeding with the annealing treatment, more of the surface is covered by connected long nanostructures (Figure 3c) until, after 30 min, completely formed nano-islands are created (Figure 3d). The crystalline nature of the dewetted islands is proven, as is clear from the cross-view SEM images reported in Figure 3a and Figure 4, where equilibrium facets typical of crystals can be recognized.

Ge content and strain before and after annealing were investigated by XRD and Raman spectroscopies. XRD shows that the epitaxial Si_1−x_Ge_x_layer is initially fully strained with *x* = 0.29. The SiGe peak is very narrow in the *q*_‖_ direction and vertically aligned with the Si device layer both in (004) and in (224). No appreciable strain relaxation occurs up to 15 min, but some broadening of the SiGe peak occurs after 15 min due to the introduction of the first defects. At 20 min, the formation of a low-strain Si_1−x_Ge_x_ layer of *x* = 0.21 can be seen due to intermixing with the Si device layer, while some defective but (almost) strained *x* = 0.29 Si_1−x_Ge_x_remains, but at 30 min the whole film is *x* = 0.21 with low strain. The peak is very broad due to the 3D nature of the film and the density of defects associated with relaxation, and the low degree of strain is evident from that fact that q_⊥_/4 ≈ *q*_‖_/2√2, as to be expected for cubic crystals in the (224) reflection.

The shift in the position of each Raman peak related to the nanostructures formed during the annealing treatment is to be ascribed to the different Ge content of the alloy and the presence of compressive or tensile strain, since both phenomena strongly affected the optical phonon modes. Pezzoli et al. [58] proposed the following equations to investigate both the Ge content and strain of a continuous film of Si_1−x_Ge_x_alloy, where x represents the germanium content and ε is the biaxial in-plane strain:

(1) $$\omega_{Si-Si}\left(x,\varepsilon \right)=520.5-66.9x-730\varepsilon$$

(2) $$\omega_{Si-Ge}\left(x,\varepsilon \right)=400.1+24.5x-4.5x^{2}-33.5x^{3}-570\varepsilon$$

(3) $$\omega_{Ge-Ge}\left(x,\varepsilon \right)=280.3+19.4x-430\varepsilon$$

Since the Ge-Ge peak in our samples is very weak, we only used Equations (1) and (2) to evaluate the Ge content and the strain. The results are reported in Table 1.

As can be observed, a good agreement between XRD and Raman results is obtained. The higher discrepancy obtained for the 20 min and 30 min samples is justified, since the strain-shift coefficients in Equations (1)–(3) refer to biaxial strain in continuous films, not for the three-dimensional morphology typical of dewetted systems. However, in the case of dewetted systems, the strain is in any case reduced such that the relevance of the strain shift is minor. A trend is clearly observed.

It is worth noting that while global strain relaxation trends are clearly observed during dewetting, local strain may vary among nanoislands of different sizes. Smaller islands may remain coherent with the underlying Si due to lower total strain energy and the possibility of elastic expansion, while larger islands may accommodate strain more effectively through interdiffusion or defect generation. Spatially resolved strain mapping would be required to quantify this effect and could be a valuable direction for future studies.

To conclude, let us summarize and correlate the results observed by SEM, AFM, XRD and Raman. From the SEM and AFM morphological investigation, it is possible to conclude that the onset of void nucleation starts after 10 min annealing, when the film roughness increases. This evidence is considered the very beginning of the SSD process. The opening of voids, with small finger formation and the creation of elongated nanostructures, starts after 15 min. After 20 min, the surface is completely covered by the elongated structures, which are still connected, and in 30 min the dewetting is complete.

Complementary insights from XRD and Raman analyses reveal that both Ge content decrease and strain relaxation begin after 15 min and they are particularly appreciable only at about 20 min.

Taken together, these observations indicate that dewetting is the first process to initiate during thermal treatment. The subsequent interdiffusion of Si from the SOI layer and the relaxation of the film occur at later stages, suggesting that morphological changes (accompanied by the motion of matter) precede and possibly promote both compositional redistribution and relaxation.

Finally, although higher Ge content would be preferable for infrared applications, here, the choice of a Si_0.71_Ge_0.29_ alloy enables a balanced investigation into strain relaxation, interdiffusion, and dewetting dynamics, making it ideal for this fundamental, proof-of-concept study.

While the present study focuses on the fundamental understanding of strain relaxation and interdiffusion during spontaneous dewetting, control over the spatial distribution and density of the resulting islands is essential for device integration. Although not addressed here, such control can be achieved by tailoring annealing temperature, time, and film thickness, as already demonstrated [6,8,29,50]. Additionally, patterning the film before annealing, for instance by EBL, can guide the dewetting process and enable ordered nanoisland array fabrication, as reported in [17,18,25,49].

## Figures and Tables

**Table 1:** Results of XRD and Raman data analysis. x indicates the Ge content; ε is the in-plane strain. For C, two regions in the RMSs have been identified, but the Raman measurement only identifies the intermixed low-strain material.

Sample	XRD Results	Raman Results
As-grown	x=0.29	x=0.27
	ε=−1.10	ε=−1.10
A 10 min	x=0.29	x=0.27
	ε=−1.10	ε=−1.10
B 15 min	x=0.29	x=0.27
	ε=−1.10	ε=−1.00
C 20 min	x=0.28; 0.21	x=0.21
	ε=−0.90;−0.22	ε=−0.17
D 30 min	x=0.20	x=0.20
	ε=−0.05	ε=−0.03

## Data Availability

Data are available at the following DOI: 10.5281/zenodo.15668189.
